# Leg Muscle Activity and Joint Motion during Balance Exercise Using a Newly Developed Weight-Shifting-Based Robot Control System

**DOI:** 10.3390/ijerph20020915

**Published:** 2023-01-04

**Authors:** Go Yamako, Kirari Ito, Takanori Muraoka, Etsuo Chosa

**Affiliations:** 1 Department of Mechanical Engineering, Faculty of Engineering, University of Miyazaki, Miyazaki 889-2192, Japan; 2 Department of Medicine of Sensory and Motor Organs, Division of Orthopedic Surgery, Faculty of Medicine, University of Miyazaki, Miyazaki 889-1601, Japan

**Keywords:** balance exercise, center of pressure, postural control, ankle strategy, weight shift, falls, robot, rehabilitation, locomotion robot

## Abstract

A novel and fun exercise robot (LOCOBOT) was developed to improve balance ability. This system can control a spherical robot on a floor by changing the center of pressure (COP) based on weight-shifting on a board. The present study evaluated leg muscle activity and joint motion during LOCOBOT exercise and compared the muscle activity with walking and sit-to-stand movement. This study included 10 healthy male adults (age: 23.0 ± 0.9 years) and examined basic LOCOBOT exercises (front–back, left–right, 8-turn, and bowling). Electromyography during each exercise recorded 13 right leg muscle activities. Muscle activity was represented as the percentage maximal voluntary isometric contraction (%MVIC). Additionally, the joint motion was simultaneously measured using an optical motion capture system. The mean %MVIC differed among LOCOBOT exercises, especially in ankle joint muscles. The ankle joint was primarily used for robot control. The mean %MVIC of the 8-turn exercise was equivalent to that of walking in the tibialis anterior, and the ankle plantar flexors were significantly higher than those in the sit-to-stand motion. Participants control the robot by ankle strategy. This robot exercise can efficiently train the ankle joint muscles, which would improve ankle joint stability.

## 1. Introduction

Postural control is a complex process based on the dynamic interaction of the sensory systems (visual information, the vestibular system, and the somatosensory system) and motor systems (neuromusculoskeletal) [1]; it is the act of maintaining, achieving, or restoring a state of balance in posture or any activity [2]. Deterioration or impairments of the postural control systems result in a weakened ability to maintain balance and an increased risk of falls and fall-related injuries. Nearly 30% of people aged ≥ 65 years fall at least once per year [3,4], and approximately 20% of the falls have severe consequences, including serious injuries and fractures, some of which lead to immobility or death [3,5]. Overall, 55% of the stroke population experiences one or more falls, 42% experience repeated falls, and 54% experience near-falls [6]. Children with diplegic cerebral palsy have a limited range of the motor skills required for postural balance, increasing the risk of falls and fear of falling [7]. Impairments to the balance and postural stability likely contribute to the increased risk of falls and fractures in patients with Parkinson’s disease [8]. Moreover, the maintenance of balance is not only important in daily activities of living, but also in competitive sports activities [9].

Balance training in fall-risk populations can decrease the incidence of falls and improve postural control. However, general exercise consists of repetitive motions with long practice times that are difficult to continue as routine tasks because individuals need patience and motivation. Video-game-based exercises are becoming a popular technology for balance training to overcome this issue. These exercise can motivate people to practice, and by performing dual tasks, users can train both cognitive and motor skills [10]. Nintendo Wii Fit is one of the most famous video game systems; it consists of exercise-based game software, the Wii console and the Wii Balance Board (WBB). The WBB can measure the center of pressure (COP) on the board and is used for controlling objects on a monitor. Typical video game tasks include COP control based on weight-shifting on the WBB. This system applies to rehabilitation for assessing and training individual balance ability [11,12]. Active weight-shifting used to control a video game, such as the Wii Fit, is considered to be an effective treatment for improving functional and dynamic balance in older adults [13,14] and people with stroke [15], cerebral palsy [16], and Parkinson’s disease [17]. Dynamic COP control and its visual feedback have the potential to improve balance ability for preventing falls. However, while playing a video game, it is difficult to feedback the COP direction in a real space, because the monitor is generally set vertically on a floor, whereas the object represented by the COP is in a virtual space. Users may lose balance because they do not recognize the COP position on the base of support.

Thus, we have developed a novel weight-shifting-based robot control system (LOCOBOT^®^, locomotion robot) to provide fun and motivation in balancing exercises. The LOCOBOT is a system where a spherical robot moves on a flat floor in the COP direction in a real space. This study aimed to clarify the characteristics of leg muscle activity and joint motion during balance exercise using the LOCOBOT and evaluate differences in muscle activity to basic daily living movements, including walking and sit-to-stand.

## 2. Materials and Methods

### 2.1. Weight-Shifting-Based Robot Control System

**Figure 1:**
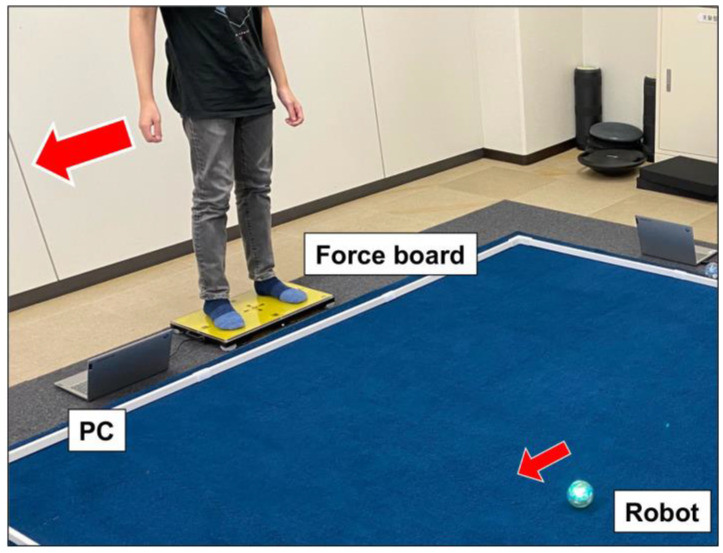
Newly developed weight-shifting-based robot control system (LOCOBOT, locomotion robot). Users stand on a force board and can move the spherical robot freely on a floor through weight shifting. The laptop PC is connected to the force board and measure forces of the loadcell placed on each corner and the center of pressure (COP) is calculated; then, the robot is wirelessly moved in the direction based on COP.

LOCOBOT (LOCOBOT Inc., Miyazaki, Japan) can control a spherical robot (Sprk+, Shpero Inc., Boulder, CO, USA) based on weight-shifting on a force board (Figure 1). The robot moves on a floor in the COP direction on a force board (50 × 32 cm), which has four load cells at each corner of the board. The voltage signal of each load cell is recorded using a Wheatstone bridge amplifier and analog-to-digital converter connected to a laptop PC at 100 Hz. Then, the vector of the COP is calculated from the four loads and the moving direction is wirelessly sent to the robot using Bluetooth. The moving speed of the robot changes according to the norm of the COP vector. A constant speed of 0.5 m/s (normal speed) was used in this study. The robot can stop still while the COP is within the center circle (diameter of 6 cm) of the board. Users need to perform active COP control through postural changes on the board to freely move the robot, and receive visual COP feedback. Users can enjoy a sense of synchronization with the robot and enjoy various games, such as track races, bowling, and tag in real space, together with other users.

### 2.2. Participants

This cross-sectional study included 10 healthy male adults (age: 23.0 ± 0.9 years, mass: 64.6 ± 14.9 kg, height: 1.74 ± 0.04 m). None of the participants had any known musculoskeletal or neuromuscular conditions. This study was approved by the Research Ethics Committee of the Faculty of Medicine, University of Miyazaki (reference number: O-0832) and was conducted following the Declaration of Helsinki. All participants provided written informed consent before study enrollment.

### 2.3. LOCOBOT Exercise

**Figure 2:**
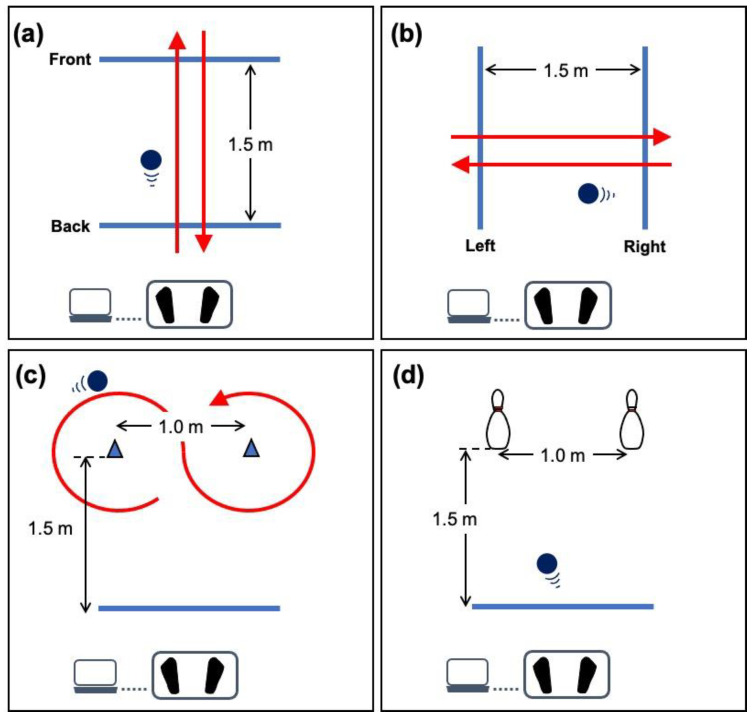
Basic balance exercises using LOCOBOT. Four typical exercises, (**a**) front–back, (**b**) left–right, (**c**) 8-turn, and (**d**) bowling, were examined. Each exercise was repeated five times continuously.

This study evaluated muscle activity and joint motion during exercise using LOCOBOT (front–back, left–right, 8-turn, and bowling) (Figure 2). Participants were instructed to stand in an upright posture on the board with feet shoulder-width apart. Participants move the robot front and back across two lines 1.5 m apart by weight-shifting on the board in the front–back exercise (Figure 2a). Participants move the robot left and right across the two lines at 1.5 m apart in the left–right motion (Figure 2b). Participants were instructed to move the robot to draw a figure of 8 in the 8-turn exercise (Figure 2c). Participants knocked down two pins at 1.0 m apart in the bowling exercise (Figure 2d). Each LOCOBOT exercise was repeated five times. Participants were allowed to practice sufficiently before measurements. Additionally, muscle activity and movement for walking and sit-to-stand movement were measured to compare LOCOBOT exercise with a basic ADL motion. Participants walked 9 m at normal speed in the lab to measure walking. The sit-to-stand motion was repeated five times, and the last cycle was used for analysis. Participants were given 5 min of rest between each measurement.

### 2.4. Electromyography (EMG) and Processing

During the LOCOBOT balance exercise and basic daily movements, EMG signals were recorded from 13 right-side leg muscles: tibialis anterior, peroneus longus, soleus, gastrocnemius lateralis, gastrocnemius medialis, vastus lateralis, vastus medialis, rectus femoris, biceps femoris, semitendinosus, adductor magnus, gluteus maximus, and gluteus medius using a telemetric EMG system (Telemyo 2400T G2 EM-602, Noraxon Inc., Scottsdale, AZ, USA) at 1500 Hz. Participants’ skin was shaved and cleaned with isopropyl alcohol before data collection to minimize impedance. Electrodes with a 2 cm inter-electrode distance (BlueSensor M-00-S, Ambu A/S, Ballerup, Denmark) were attached to the skin surface following the Surface EMG for noninvasive Assessment of Muscles (SENIAM) recommendations [18]. The raw EMG signal was filtered through a band pass filter between 10 and 750 Hz using a fourth-order Butterworth filter. The root mean square values of the EMG signal were calculated for consecutive segments of 100 ms and processed using MyoResearch XP software (Noraxon Inc., Scottadale, AZ, USA). The maximal voluntary isometric contraction (MVIC) was measured for all muscles to normalize the EMG signal (%MVIC) [19,20,21,22,23,24]. The mean %MVIC was used to evaluate muscle activity during each motion.

### 2.5. Motion Tracking and Joint Movement Analysis

Joint motions during the LOCOBOT exercise and basic movements were simultaneously recorded with EMG measurements using a camera-based optical motion capture system (Vicon Nexus, Vicon Motion Systems, London, UK) with 13 infrared cameras (Vantage-V8, MX T20-S, Vicon Motion Systems) at a sampling rate of 100 Hz and with six force platforms. Thirty-five reflective markers (14 mm in diameter) were attached to the participants, according to the Plug-in-Gait marker protocol [25,26,27]. Trajectories of markers were converted to kinematic variables of the hip, knee, and ankle joints. Range of motion (ROM) for the hip (flexion–extension), knee (flexion–extension), and ankle (dorsiflexion–plantarflexion) were analyzed in each motion. Additionally, total angular distance (TAD) was calculated to evaluate the joint movement during each LOCOBOT exercise. The TAD was defined as the moving distance of angular displacement of the joint divided by time (unit: deg/s) during exercise.

### 2.6. Statistical Analysis

In this study, we examined the differences in mean %MVIC among LOCOBOT exercises (front–back, left–right, 8-turn, and bowling). Then, the mean %MVIC of the 8-turn exercise, which required the most difficult COP control, was compared with that of walking and sit-to-stand motions. We also examined differences in the ROM and TAL among joints during each LOCOBOT exercise. All data are presented as the mean  ±  standard deviation. Multiple comparison tests with Bonferroni correction following the Kruskal–Wallis test were performed to compare the mean %MVIC. Tukey’s honestly significant difference test following one-way analysis of variance was performed to compare the ROM and TAL. Normality and variance homogeneity of the data were evaluated using the Shapiro–Wilk test and Levene’s test. All statistical analyses were performed using Statistical Package for the Social Sciences version 28.0 (IBM SPSS, Chicago, IL, USA). A *p*-value of <0.05 was considered statistically significant. Power analysis was performed to determine the sample size using G*Power 3 (version 3.1.9.6) [28]. The mean %MVIC of the tibialis anterior was selected as the primary parameter to calculate the sample size for comparison among each exercise. Considering the effect size of 0.85, which was determined from the mean %MVIC of the tibialis, a power of 0.95, and alpha level of 0.05, a minimum sample size of 7 participants per group was required. Thus, the sample size of this study was set to 10.

## 3. Results

### 3.1. Muscle Activity

#### 3.1.1. Comparison among LOCOBOT Exercises

Muscle activity (mean %MVIC) differed depending on the LOCOBOT exercise (Table 1). Significant differences were observed in the tibialis anterior, soleus, gastrocnemius lateralis, rectus femoris, vastus lateralis, and vastus medialis. However, no significant differences were found in the hip joint muscles.

The mean %MVIC of the tibialis anterior was significantly greater in the front–back exercise than that in left–right (*p* = 0.005) and bowling (*p* < 0.001) exercises. The 8-turn exercise exhibited significantly greater muscle activity than the bowling motion (*p* = 0.002).

The mean %MVIC for knee extension muscles (vastus lateralis, vastus medialis, and rectus femoris) was relatively greater in the front–back exercise than that of left–right exercise, but with no significant differences (*p* = 0.124 for vastus lateralis, *p* = 0.077 for vastus medialis, and *p* = 0.056 for rectus femoris). The mean %MVIC for rectus femoris was significantly greater in the front–back motion than that of bowling motion (*p* = 0.044). No significant differences were found in the mean %MVICs between the front–back and 8-turn exercises (*p* = 1.000 for vastus lateralis, *p* = 1.000 for vastus medialis, and *p* = 1.000 for rectus femoris).

#### 3.1.2. Comparison of 8-Turn LOCOBOT Exercise to Walking and Sit-to-Stand Motion

Muscle activity (mean %MVIC) differed among movements (Table 2). Significant differences were found in the ankle dorsiflexion muscle (tibialis anterior), ankle plantar flexion muscles (peroneus longus, soleus, gastrocnemius lateralis, and gastrocnemius medialis), knee extension muscles (vastus lateralis, vastus medialis, and rectus femoris), and knee flexion muscle (semitendinosus). No significant difference was observed in the hip joint muscles (adductor magnus, gluteus maximus, and gluteus medius).

No significant difference was found in the mean %MVIC of the tibialis anterior between 8-turn and sit-to-stand movement for ankle joint muscles (*p* = 0.099). In contrast, sit-to-stand exhibited a significantly greater muscle activity of the tibialis anterior compared with walking (*p* = 0.007). The mean %MVIC of the 8-turn exercise was significantly greater than that of sit-to-stand at soleus (*p* = 0.047), gastrocnemius lateralis (*p* = 0.023), and gastrocnemius medialis (*p* = 0.027). In contrast, no significant difference was found in the mean %MVIC between 8-turn and walking motions (tibialis anterior: *p* = 1.000, peroneus longus: *p* = 1.000, soleus: *p* = 0.893, gastrocnemius lateralis: *p* = 0.443, and gastrocnemius medialis: *p* = 0.364).

The mean %MVIC of sit-to-stand was significantly greater than that of walking in the rectus femoris (*p* = 0.002), vastus lateralis (*p* = 0.004), and vastus medialis (*p* = 0.01) for knee extension muscles. However, no significant differences were observed between 8-turn and sit-to-stand in the rectus femoris (*p* = 0.511), vastus lateralis (*p* = 0.759), and vastus medialis (*p* = 0.112).

### 3.2. Joint Motion during LOCOBOT Exercise

#### 3.2.1. ROM

ROM differed among joints for each LOCOBOT exercise (Table 3). A significant difference was found in ROM for left–right and 8-turn exercises. ROM of the ankle joint was significantly greater than that of the hip joint in the left–right exercise (*p* = 0.005). ROM of the ankle joint was significantly greater than that of the knee joint in the 8-turn exercise (*p* = 0.044).

#### 3.2.2. TAD

TAD differed among joints for the left–right exercise (Table 4). TAD of the ankle joint was significantly larger than that of the hip joint. TAD of the ankle joint was relatively greater than that of the knee and hip joint, but with no significant difference, for other LOCOBOT exercises.

## 4. Discussion

The present study aimed to demonstrate the characteristics of muscle activity and joint movement in balance exercise using a newly developed weight-shifting-based robot system and to compare those with basic dairy motions.

### 4.1. Muscle Activity and Joint Movement during LOCOBOT Exercise

This study revealed various muscle activities among LOCOBOT exercises, especially in ankle joint muscles. In the tibialis anterior, 8-turn and front–back exercises exhibited greater activity than left–right and bowling exercises. The tibialis anterior is the primary muscle activating ankle dorsiflexion that could maintain toe clearance during the swing phase in walking; therefore, balance exercise interventions focus on this muscle for fall prevention. Additionally, both ROM and TAD were higher for the ankle joint than for the knee and hip joints. These results indicate that the ankle joint is primarily used for the operation of robots by COP control, named the “ankle strategy”, which is explained by an inverse pendulum model on balance control in an upright posture [29]. Previous studies showed that during perturbed and unperturbed balance while standing, the most prevalent control strategy was an ankle strategy [30].

The tibialis anterior showed high activity when the COP was posterior (moving the robot backward) during the LOCOBOT exercise. This is because dorsiflexion torque of the ankle joint generated by the tibialis anterior supports the body so that it does not fall backward. The tibialis anterior activity of left–right and bowling exercises was lower than that of 8-turn and front–back exercises. This is because there was less need to move the COP posteriorly. Muscle activity of the peroneus, soleus, lateral gastrocnemius, and medial gastrocnemius muscle was high, and ankle plantarflexion torque was exerted to prevent the body from falling forward when the COP was anterior (moving the robot forward). Therefore, the activity of the ankle plantar flexor muscles was high in the bowling exercise, in which moving the robot anteriorly is dominant. Developing an effective exercise program that includes various movements of the COP using LOCOBOT is important for stimulating various muscles because the muscle activity differs depending on the exercise.

### 4.2. Comparison of 8-Turn LOCOBOT Exercise with Walking and Sit-to-Stand

We compared the mean muscle activity in the 8-turn exercise, which requires advanced COP control in LOCOBOT exercises, with basic ADL, including walking and sit-stand movements. The tibialis anterior activity of the 8-turn exercise was the same level as that of the walking exercise. The comparison of the muscle activity in the 8-turn exercise with that of the sit-to-stand movement revealed no significant difference in the tibialis anterior and peroneus longus, but the soleus, gastrocnemius lateralis, and gastrocnemius medialis showed significantly higher muscle activity. The ankle dorsiflexion torque generated by the tibialis anterior acted strongly to prevent the body from falling backward at the initial phase of movement during standing from a chair; thus, the tibialis anterior activity was greater than that during walking. Conversely, the ankle plantarflexion muscles are less necessary during standing from a chair; thus, the muscle activity was lower than that during walking. Knee extensor muscles are significantly greater during standing from a chair than during walking. This is because the extensor muscles are necessary to lift the body upward in standing-up motion. Therefore, the muscle activity of the 8-turn LOCOBOT exercise is equivalent to that of walking in the tibialis anterior, and the ankle plantar flexors are significantly higher than those of the sit-to-stand motion. These results indicate that LOCOBOT exercise based on COP control can efficiently train the ankle joint muscles, which would contribute to ankle joint stability improvement. In fact, COP control exercise using Wii Fit improved the muscle activities of the tibialis anterior and gastrocnemius medialis in healthy adults [31]. LOCOBOT is now clinically used for balance training after total hip arthroplasty, and LOCOBOT exercises improve the weight-bearing ratio between operated and non-operated sides during standing in a short period postoperatively [32].

LOCOBOT can also adjust the difficulty level of COP control according to the user’s balance ability. For example, making the contact surface of the foot unstable and increasing the difficulty of COP control is possible by placing a balance pad on the force board. Additionally, muscle activity in the lower extremity muscles, especially in knee extensors, could be increased by controlling the robot in a squat posture. Elderly people who are at risk of falling could control the robot using a support such as a cane or while sitting on a chair.

One of our study limitations was that the participants were only young male adults. The COP control manner by weight shifting may vary depending on gender, age, and disease history. Previous studies have demonstrated that postural control strategies differ between older and young adults [33], and between genders [34].

## 5. Conclusions

In conclusion, we developed a novel COP-based exercise robotic system for improving balance for postural control and demonstrated the characteristics of leg muscle activity and joint motion in healthy young male adults. The LOCOBOT exercise may be a novel and fun way to efficiently improve ankle stability by dynamic COP control. Further intervention studies are needed to examine the effect of the LOCOBOT exercise on the recovery of balance ability of older people and patients with stroke, cerebral palsy, and Parkinson’s disease.

## Figures and Tables

**Table 1:** Mean %MVIC of leg muscles during LOCOBOT exercises.

Muscle	Front–Back	Left–Right	8-Turn	Bowling	*p*-Value (Kruskal–Wallis Test)
Tibialis anterior	8.3 ± 3.2	2.8 ± 2.1	6.4 ± 2.1	1.5 ± 0.7	<0.001 **
Peroneus longus	4.7 ± 1.6	3.5 ± 1.4	4.2 ± 1.3	5.4 ± 1.8	0.104
Soleus	7.1 ± 2.6	6.3 ± 2.2	6.3 ± 2.2	9.5 ± 2.9	0.038 *
Gastrocnemius lateralis	2.6 ± 0.8	2.6 ± 1.9	2.3 ± 0.7	4.6 ± 2.1	0.003 **
Gastrocnemius medialis	5.8 ± 3.3	4.5 ± 2.8	5.1 ± 2.9	8.1 ± 6.2	0.709
Vastus lateralis	11.8 ± 7.5	6.8 ± 7.6	10.8 ± 8.0	6.4 ± 7.8	0.017 *
Vastus medialis	5.3 ± 3.8	2.8 ± 3.5	4.8 ± 3.8	3.1 ± 3.9	0.025 *
Rectus femoris	4.7 ± 2.4	2.4 ± 2.1	4.1 ± 2.8	2.3 ± 2.4	0.013 *
Biceps femoris	2.4 ± 1.8	2.3 ± 1.7	3.1 ± 1.8	2.4 ± 2.3	0.520
Semitendinosus	6.0 ± 2.0	4.7 ± 2.6	5.9 ± 2.5	6.6 ± 3.2	0.278
Adductor magnus	2.2 ± 1.4	1.1 ± 0.6	2.1 ± 1.5	1.4 ± 0.7	0.132
Gluteus medius	1.6 ± 1.1	3.1 ± 1.3	2.5 ± 1.3	2.4 ± 1.2	0.062
Gluteus maximus	1.1 ± 0.7	1.2 ± 0.4	1.4 ± 0.6	1.1 ± 0.5	0.657

* *p* < 0.05, ** *p* < 0.01. Abbreviation: MVIC, maximal voluntary isometric contraction.

**Table 2:** Comparison of mean %MVIC in 8-turn LOCOBOT exercise with that of waking and sit-to-stand movement.

Muscle	8-Turn	Walking	Sit-to-Stand	*p*-Value (Kruskal–Wallis Test)
Tibialis anterior	6.4 ± 2.1	5.0 ± 2.0	9.4 ± 3.8	0.007 **
Peroneus longus	4.2 ± 1.3	6.1 ± 2.8	2.7 ± 1.1	0.005 **
Soleus	6.3 ± 2.2	7.9 ± 3.7	3.4 ± 2.0	0.002 **
Gastrocnemius lateralis	2.3 ± 0.7	3.9 ± 1.9	1.0 ± 0.5	<0.001 **
Gastrocnemius medialis	5.1 ± 2.9	8.1 ± 3.0	1.3 ± 1.0	<0.001 **
Vastus lateralis	10.8 ± 8.0	4.7 ± 2.8	12.2 ± 5.1	0.005 **
Vastus medialis	4.8 ± 3.8	3.2 ± 2.1	9.5 ± 5.2	0.01 *
Rectus femoris	4.1 ± 2.8	1.8 ± 1.1	7.3 ± 4.2	0.003 **
Biceps femoris	3.1 ± 1.8	2.2 ± 1.4	1.4 ± 1.0	0.061
Semitendinosus	5.9 ± 2.5	3.0 ± 1.5	1.4 ± 0.7	<0.001 **
Adductor magnus	2.1 ± 1.5	2.8 ± 2.1	2.3 ± 1.8	0.745
Gluteus medius	2.5 ± 1.3	2.7 ± 1.9	2.0 ± 1.1	0.784
Gluteus maximus	1.4 ± 0.6	1.4 ± 1.0	2.0 ± 1.1	0.343

* *p* < 0.05, ** *p* < 0.01, Abbreviation: MVIC, maximal voluntary isometric contraction.

**Table 3:** Range of motion (unit: deg) of each joint during LOCOBOT exercises.

Exercise	Ankle Joint	Knee Joint	Hip Joint	*p*-Value (One-Way ANOVA)
Front–back	26.2 ± 12.7	20.9 ± 16.3	23.8 ± 15.8	0.737
Left–right	24.4 ± 11.4	17.3 ± 9.7	10.5 ± 5.0	0.008 **
8-turn	45.1 ± 22.0	24.6 ± 13.8	26.6 ± 17.5	0.032 *
Bowling	24.5 ± 10.3	18.3 ± 8.9	19.3 ± 7.7	0.282

* *p* < 0.05, ** *p* < 0.01. Abbreviation: ANOVA, analysis of variance.

**Table 4:** Total angular distance (unit: deg/s) among joints during LOCOBOT exercises.

Exercise	Ankle Joint	Knee Joint	Hip Joint	*p*-Value (One-Way ANOVA)
Front–back	26.2 ± 12.7	20.9 ± 16.3	23.8 ± 15.8	0.075
Left–right	24.4 ± 11.4	17.3 ± 9.7	10.5 ± 5.0	0.007 **
8-turn	45.1 ± 22.0	24.6 ± 13.8	26.6 ± 17.5	0.18
Bowling	24.5 ± 10.3	18.3 ± 8.9	19.3 ± 7.7	0.193

** *p* < 0.01. Abbreviation: ANOVA, analysis of variance.

## Data Availability

Data are available on request due to restrictions, e.g., privacy or ethical.
